# Condensations from Late Editorials in the Medical Press

**Published:** 1882-01

**Authors:** 


### Condensations from Late Editorials in the Medical Press.

PSYCHOLOGY IN PHYSIC. — In an article on "Doctor and Patient," our versatile contemporary, the *Spectator*, lays it down as an axiom that "to be a really good physician, a man must be a psychologist;" by which he means that the inner nature, or heart, or soul—call it what we will—of the patient must be taken into account in every diagnosis and in every scheme of treatment, as part of the case, and of the subject as a whole. This is undoubtedly true. Whatever may be the causal relations between mind and body, the mental part of man's being is not less real than the material, and it plays an equally realistic part in the production of disease, the constitution of morbid states, and resistance to remedies. The practitioner who should leave the mind out of consideration in the study of disease in the concrete, or disregard it in the plan of treatment he adopts, would be closing his eyes to half the task he undertakes, and throwing away half the means and power by which it is to be accomplished. —*London Lancet*, *November*.

VIVISECTION.—Three of the addresses delivered at the late meeting of the International Medical Congress dealt with the subject of the value of experiments on living animals. Each was written by a master hand, fully competent for the task, and presented the arguments in favor of such experiments from a different point of view. Professor Fraser demonstrated how necessary such experiments are to scientific investigation in pharmacology, and illustrated from his own experience how completely the present act for the Prevention of Cruelty to

Animals has stopped progress in this direction in our own country. M. Simon, in his address, showed the good results such investigations have already rendered to preventive medicine, in particular, and pointed out the abundant harvest yet to be reaped in the same fields of labor. Professor Virchow made a powerful appeal for perfect freedom in scientific research, and conclusively showed that our present knowledge of life is founded on experiment, and that the only reliable and practicable means of increasing and perfecting it is also experiment.—*Idem.*

BACON AND MICROSCOPE.—The organization of laboratories in which foreign bacon shall be examined is announced. A school, or rather a course of micrography and helminthology, will be organized, with the aim to create in France an army of inspectors, whose mission shall be to protect us against the invasions of parasites through the most useful aliments. (The editor reviews here, in a sarcastic tone, the trichinosis scare, and quotes the *Marseille Médical*): "From Feb. 24 to May 28, 1881, a special commission, under the direction of M. Marroin, made thirteen examinations, looking over 5,229 specimens of bacon. 24,839 histological preparations were made. Among these 70 were found to contain trichinæ, or an average of $\frac{1}{3}$ per cent. These represented 1,229 barrels, containing hams, salt pork, ribs and steaks. About 9,000 barrels came under the supervision of that commission, giving an average of 0.77 trichinosed barrels in 100; or about 0.031 of trichinosed pieces in a hundred. The examinations made at Havre failed to discover any trichinæ at all.

"We must acknowledge this, that the meat exported from America presents a pretty good appearance, and could not be that of diseased animals, if we pay attention to the quantity of adipose tissue which it contains."

Iu view of these results, which do not render it possible to affirm in every case the wholesome nature of the meat examined, it seems that England was wise in abstaining from any coercive measures, adopting the principle that the best preventive of trichinosis consists in a good roasting or boiling of the suspected meat, and this is also a general rule in this country.—*Union Médicale et Scientifique du Nord Est, Oct.*

DOES VACCINATION PROTECT?—It is becoming somewhat tiresome to have to reiterate the truths about vaccination. And it will sometimes occur to us that the best way, after all, would be to leave the question alone, and let the people find out the facts for themselves. Why should the doctor worry himself? If the people do not and will not believe in vaccination, why not drop our quills, and, having vaccinated ourselves and families, let the disease work away in its old-fashioned seventeenth century style. In 1721, half of the city of Boston lay sick with the small-pox. Would not the return of such a visitation be better than any pamphlets or statistics?

We present here a very few of the facts and figures upon which this universal agreement is based. It should be remembered, however, that aside from statistical proof, nearly every physician, at some time during his life, gets personal evidence of the efficacy of Jenner's discovery.

Previous to the introduction of vaccination, the annual mortality from small-pox throughout Europe was about three thousand to every million inhabitants. During the forty years subsequent, the mortality from the same cause was reduced in Sweden to 158 per million; in Westphalia to 114; in Bohemia, Moravia, and Austrian Silesia to about 200; in Copenhagen to 286; in Berlin to 176; in England to 200.

In 1853, the Epidemiological Society received two thousand letters from medical men, affirming their belief, from personal experience, in the protective power of vaccination. Dr. Simon obtained similar answers from five hundred and forty eminent English and foreign physicians, out of five hundred and forty-two to whom letters were addressed.

One observer, Marshall, has shown that among 757 persons exposed to small-pox, 231 had been vaccinated, and of these latter only 27 took the disease. All the remainder, except seven, were infected. In 1871, an anti-vaccination excitement was fomented in England, and Parliament undertook to investigate the question. A large committee was appointed, and testimony taken from every quarter. The result was an overwhelming refutation of the points claimed by the anti-vaccinationists.

In the Franco-Prussian war, an epidemic of small-pox arose among the unvaccinated inhabitants of Brittany. It spread among the French soldiers, and destroyed 23,000 of them. The Prussian army, which was frequently and extensively exposed, and was larger than the French, lost only about 250 persons by the disease. The Prussian army was thoroughly vaccinated, the French was not. In the whole Prussian army for twenty years, though often exposed, only four fatal cases of small-pox occurred among the revaccinated.

There are a few things which have to be borne in mind when arguing for vaccination:

1. Vaccination is protective only when the virus is good, and has entered and affected the system.

2. There is a very small number of persons who will take small-pox if exposed, whether they have been vaccinated or not. If vaccinated, however, most of these will have the disease less severely.

3. Vaccination protects in most cases only for a certain period of years, and revaccination is necessary.

4. Vaccine lymph may possibly deteriorate after passing through the human system many times. This deterioration of humanized lymph has probably taken place in England, and perhaps elsewhere in Europe.

5. Small-pox varies in malignancy with the epidemic. There is no evidence, however, to prove that small-pox is any less malignant now, on the whole, than it was eighty years ago.

The above facts have to be considered in passing judgment upon the so-called statistics of anti-vaccinationists.—*Medical Record.*

PRACTICE OF MEDICINE BY DRUGGISTS. — Under the care of the senior editor, lately, came a patient who had been for several weeks under treatment by a "respectable" druggist of San Francisco, on account of a real or supposed venereal affection. He had taken "eight dozen" mercurial pills, which the druggist had persisted in prescribing until a slight induration should disappear. Meanwhile, the general health of the man became more and more impaired, and finally an erythematous eruption covered the body, which induced the prescriber to shut off the mercury. There is every reason to depend on the veracity of the informant in this case. Such cases are not rare. Indeed, they are so frequent as to have created a feeling on the part of the physicians against apothecaries which should not exist, and which is prejudicial to the interest of both. There should be some way of discriminating against druggists who perpetrate such frauds—for fraud it is, particularly in view of the law which imposes a heavy penalty on the practice of medicine without a license. We propose that every local and medical society keep a record, in which the names of all who are thus guilty shall be recorded—the members of the society to report every case of the kind coming under their notice. Perhaps a black list of this kind would deter some apothecaries from traveling in the "ways which are dark."—*Pacific Med. and Surg. Journal.*

HOSPITALS.—The most frequent error made in regard to hospitals, is that these institutions are the creation of Christianity, and that to its beneficent functions solely has the infirmity and the disease of the world been indebted for such charitable relief. The most distinguished writers and speakers have, before the public, eulogized these noble institutions, and ascribed their creation and multiplication to the representatives of Christianity. Mr. Huxley refutes this popular and wide-spread fallacy, and ascribes the first existence of hospitals to the very morning of medicine; to the days of Æsculapius, and the Æsclepiades.

Mr. Huxley does not hesitate to attribute to this remote era the first creation of the hospital, and he gives such proof that no one can doubt his accuracy or fidelity. It is remarkable, however, that this great teacher did not go still farther back into the dawn of history, and show that, centuries before the Christian era, hospitals existed in India and in Egypt.—*Gaillard's Med. Journal.*

SANITARY SCIENCE JUSTIFIED BY ITS WORKS.—Those State legislatures and town governments which have not yet established boards of health, those which may be hesitating as to whether such notions pay, and certainly those politicians who may have persuaded themselves that a curtailment of the efficiency of such organizations already established, under the excuse of a little pretended economy, will in the long run be approved by the people at large; all such would do well to devote some study to the recent report of the Local Government Board, which contains, among other things, a statement of the progress of sanitary work in England and Wales. It may be useful to draw attention to the annual death-rate for some years past, as indicating the effect which recent sanitary measures would appear to have had upon the public health.

ENGLAND AND WALES.

| Annual death-rate per 1,000. | 1841–50. | 1851–60. | 1861–70. | 1871–80. |
|---|---|---|---|---|
| All causes.................. | 22.4 | 22.2 | 22.5 | 21.5 |
| Seven zymotic diseases...... | .......... | 4.11 | 4.14 | 3.36 |
| Fever....................... | .......... | 0.91 | 0.88 | 0.49 |

From the above figures it will be seen that, speaking generally, the death-rate of the country remained stationary from 1840 to 1870, but that in the period 1871–80 it fell from 22.5 (of the previous decade) to 21.5, a reduction equivalent to nearly four and one-half per cent. It may, therefore, be roughly estimated that about a quarter of a million of persons were saved from death in the ten years, 1871–80, who would have died if the death-rate had been the same as in the previous thirty years.

Comparing, then, 1861–70 with 1871–80, it will be seen from the foregoing figures, that of the entire reduction of 1.0 in the death-rate, more than three-quarters (4.14—3.36=0.78) comes under the head of "the seven zymotic (infectious) diseases;" of the diseases, that is, which are most influenced by sanitary improvements, and most amenable to control by the action of sanitary authorities.

The pecuniary gain may be thus stated: Under the inquiry as to interments, the cost of funerals—all round—was ascertained to be £5 each. The gain under that head will, therefore, be about one million by the quarter of a million of funerals saved during the last decade. The direct cost of sickness has been estimated at about £1 per case. The gain under that head during the decade will, therefore, amount to about three millions; a gain, that is to say, of medical treatment and other expenses. But the gain to the wage classes, from the saving of lost labor, will have been far greater, or upwards of thirteen millions per annum. Sanitary science, as exhibited in this statement of the Local Government Board, which is merely typical of work actually doing and work that may be done in many other parts of the world, is certainly justified of her children.—*Boston Med. and Surg. Journal.*

---

THE IMMEDIATE ARREST OF BLEEDING FROM THE NOSE.—John Kent Spender, M.D., in *British Medical Journal* says: An improved instrument is described in Mr. W. Spencer Watson's book on Diseases of the Nose and its accessory Cavities. It "consists of a gum elastic tube about five inches long, with lateral perforations near the end, and covered with thin caoutchouc membrane in the form of a spirally twisted bag for the last three or four inches of its length. To use it the membranous bag is smoothly folded over the continued tube, and the whole being oiled (diluted glycerin is better) is passed along the floor of the nares till it reaches the pharynx. The bag is now inflated * * * and if a stop-cock is fitted the air is kept in by turning it as soon as sufficient tension is obtained." The cavity of the twisted bag could be injected with water if it were desired, but I have never found this necessary. When I recollect what "bleeding from the nose" was in old days, I can not be too thankful to Dr. Rose for his simple and effective invention. To be called to an obstinate accident of this kind, especially when other medical men had failed, was enough to make one sick at heart from the possibility of adding another failure to the dreary history; and then there was the consciousness that delay might mean impaired health or even death to the victim. The victory is half won when a man is armed with an apparatus which he knows is sure to succeed; and I am now speaking of cases in which he wishes to succeed, and which are not forms of natural blood-letting to be encouraged. The object of this brief communication is to reccommend Dr. Rose's instrument for (1) facility of introduction; (2) the extent and evenness of the inflated area; and (3) the possibility of its remaining *in situ* for thirty-six or forty-eight hours, when it may be gently removed, and the hæmorrhagic nostril can be syringed with some cold astringent fluid for purposes of cleanliness and the washing away of blood *débris*.

